# The Herb–Drug Pharmacokinetic Interaction of 5-Fluorouracil and Its Metabolite 5-Fluoro-5,6-Dihydrouracil with a Traditional Chinese Medicine in Rats

**DOI:** 10.3390/ijms19010025

**Published:** 2017-12-23

**Authors:** Ju-Han Liu, Yung-Yi Cheng, Chen-Hsi Hsieh, Tung-Hu Tsai

**Affiliations:** 1 Institute of Traditional Medicine, School of Medicine, National Yang-Ming University, Taipei 112, Taiwan; hanaa77721@gmail.com (J.-H.L.); vininecheng@gmail.com (Y.-Y.C.); chenciab@gmail.com (C.-H.H.); 2 Graduate Institute of Acupuncture Science, China Medical University, Taichung 404, Taiwan; 3 Division of Radiation Oncology, Department of Radiology, Far Eastern Memorial Hospital, Taipei 220, Taiwan; 4 Faculty of Medicine, School of Medicine, National Yang-Ming University, Taipei 112, Taiwan; 5 School of Pharmacy, College of Pharmacy, Kaohsiung Medical University, Kaohsiung 807, Taiwan; 6 Department of Chemical Engineering, National United University, Miaoli 36063, Taiwan

**Keywords:** 5-fluorouracil (5-FU), 5-fluoro-5,6-dihydrouracil (5-FDHU), Xiang-Sha-Liu-Jun-Zi-Tang (XSLJZT), pharmacokinetic, herb–drug interaction, traditional Chinese medicine (TCM), HPLC–UV

## Abstract

Background: Xiang-Sha-Liu-Jun-Zi-Tang (XSLJZT) is the most common traditional formula given to colorectal and breast cancer patients in Taiwan, according to a statistical study of the National Health Insurance Research Database. 5-Fluorouracil (5-FU) is widely used as the first line of treatment for colorectal cancer. Thus, the aim of study is to investigate the pharmacokinetic interaction of XSLJZT and 5-FU. Methods: To investigate the herb–drug interaction of XSLJZT with 5-FU as well as its metabolite 5-fluoro-5,6-dihydrouracil (5-FDHU) using pharmacokinetics, a high-performance liquid chromatography (HPLC) system coupled with a photodiode array detector was developed to monitor 5-FU and 5-FDHU levels in rat blood. Rats were divided into three cohorts, one of which was administered 5-FU (100 mg/kg, iv—intravenous) alone, while the other two groups were pretreated with low and high doses of XSLJZT (600 mg/kg/day or 2400 mg/kg/day for 5 consecutive days) in combination with 5-FU. Results: The results demonstrated that 5-FU level was not significantly different between the group treated with only 5-FU and the group pretreated with a normal dose of XSLJZT (600 mg/kg/day). However, pharmacokinetic analysis revealed that pretreatment with a high dose of XSLJZT (2400 mg/kg/day) extended the residence time and increased the volume of distribution of 5-FU. No significant distinctions were found in 5-FDHU pharmacokinetic parameters at three doses of XSLJZT. Conclusions: Overall, the pharmacokinetic results confirm the safety of coadministering 5-FU with XSLJZT, and provide practical dosage information for clinical practice.

## 1. Introduction

**Figure 1:**
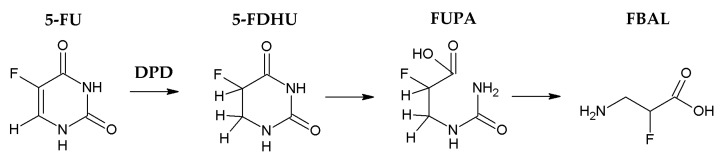
Metabolic pathway of 5-FU. 5-FU: 5-fluorouracil; 5-FDHU: 5-fluoro-5,6-dihydrouracil; FUPA: 5-fluoro-ureido-propionic; FBAL: α-fluoro-β-alanine; DPD: dihydropyrimidine dehydrogenase.

Use of the anti-metabolite 5-fluorouracil (5-FU) as a chemotherapeutic agent has maintained great clinical relevance since its introduction over 50 years ago. Certain types of cancer, including breast, esophageal, colorectal, and head and neck tumors, are treated with 5-FU-based neoadjuvant or palliative chemotherapy [1]. 5-FU and its next-generation drug, Tegafur, are particularly associated with the treatment of gastrointestinal cancers [2]. Approximately 10–30% of individuals treated with 5-FU experienced a serious life-threatening adverse effect at the standard dose [3]; these complications have been attributed to the catabolic pathway of 5-FU [4]. Over 85% of the administered 5-FU compound is degraded into 5-fluoro-5,6-dihydrouracil (5-FDHU) in the liver through a catabolic pathway mediated by dihydropyrimidine dehydrogenase (DPD) [5,6]. In addition, 5-FDHU forms 5-fluoro-ureido-propionic (FUPA) acid and α-fluoro-β-alanine (FBAL) in a two-step reaction. Only 10–20% of 5-FU is excreted in unchanged form in the urine [7,8] (Figure 1). Paolo et al. proposed that variations in 5-FU and 5-FDHU pharmacokinetics are correlated with severe toxicity in patients treated with short intravenous infusion of 5-FU [9]. It is, therefore, of vital importance to assess the plasma levels of 5-FU and 5-FDHU in order to adjust 5-FU dosing to further improve the efficacy and safety of this drug. Furthermore, because of the frequent concurrent use of anti-cancer drugs and herbal medicines, an understanding of herb–drug interactions is critical. In analytical chemistry, high-performance liquid chromatography (HPLC) is a technique applied to separate, identify, and quantify each component in a sample. Our previous studies have discussed the methods for quantification of 5-FU by using HPLC, but still, few methods could measure 5-FDHU simultaneously [10]. To date, there was no method using HPLC to monitor 5-FU and its metabolite 5-FDHU, with a traditional Chinese medicine, in a single quantitative assay.

Many cancer patients continue to seek alternative therapies for easing the adverse effects of modern chemotherapy. Traditional Chinese medicine (TCM) and herbal medicine are complementary and alternative medicines (CAM) that have been used for thousands of years in Asia, and that are increasing in popularity in the West [11]. In Europe, up to one-third of cancer patients supplement Western treatments with complementary therapy during their illness [12]. Another study, conducted in New York City, found that over half of female patients have used CAM remedies [13]. Considerable evidence suggests that herbal supplements can help to alleviate the adverse effects of chemotherapy by strengthening the immune system, improving quality of life, and delaying the progression of cancer [14,15,16,17].

According to a survey by NHIRD, XSLJZT is the most-prescribed formula for post-surgical patients with colorectal cancer and patients with breast cancer receiving chemotherapy [18,19]. XSLJZT, first described in the classic book “TCM Prescriptions by Ancient and Modern Well-Known Physicians” during the Qing Dynasty, is based on Liu-Jun-Zi-Tang (LJZT) with the addition of *Amomum villosum* and fresh costus roots (*Saussurea lappa*), and is used to promote Qi circulation [20]. XSLIZT may treat Qi deficiency resulting from dampness from the stomach and spleen by affecting the middle jioa (middle burner) to improve electrogastrogram, promote gastric emptying, and regulate gastrointestinal hormones [21]. XSLIZT is particularly effective for treating digestive diseases, such as abdominal pain, anorexia, general indigestion, nausea, vomiting, and diarrhea, and is also appropriate for the treatment of functional dyspepsia caused by multi-target therapies [22,23].

XSLJZT is a complex Chinese herbal compound mixed from eight Chinese herbs. A previous study reported that *Radix aucklandiae* (Chinese herbal name: Mu-Xiang), *Fructus amomi *(Chinese herbal name: Sha-Ren), *Rhizoma pinelliae* (Chinese herbal name: Ban-Xia), *Pericarpium citri reticulatae* (Chinese herbal name: Chen-Pi), *Radix ginseng* (Chinese herbal name: Ren-Shen), *Atractylodis rhizoma* (Chinese herbal name: Bai-Zhu), *Poria cocos (Schw.) Wolf* (Chinese herbal name: Fu-Ling), and *Glycyrrhizae radix* (Chinese herbal name: Gan-Cao) contribute to the XSLJZT formula [24]. Furthermore, some studies have reported that atractylenolide III from *Atractylodis rhizoma*, glycyrrhizic acid from *Glycyrrhizae radix* and ginsenosides from *Radix ginseng* are likely to inhibit the metabolism of coadministered medications in which the main pathway of elimination is cytochrome P450 dependent [25,26,27]. Thus, it is possible that a large enough dose of XSLJZT could suppress the expression of cytochrome P450, resulting in a decrease in the metabolic efficiency of 5-FU.

According to NHIRD studies, 5-FU and XSLJZT are commonly co-administered in cancer patients [15,16]. Besides, a survey with the PubMed database revealed no previous reports that have evaluated the pharmacokinetic interaction of 5-FU and XSLJZT. The aim of this study was to characterize the herb–drug pharmacokinetic interaction of 5-FU and XSLJZT in rats, by using high-performance liquid chromatography with photodiode array detection to assess the presence of 5-FU and its catabolic product 5-FDHU in biological samples.

## 2. Results and Discussion

### 2.1. Optimization of HPLC–UV Conditions and Sample Preparation

To optimize our experimental design, analytical columns and mobile phase compositions, such as the concentration of buffer, the buffer pH, and the percentage of organic modifiers used, must be determined. To hold the pH constant in mobile phases, the buffer solution is usually used. The buffer composed of a weak acid or weak base, combined with its conjugate base or acid in solution, that is at least partially aqueous. 5-FU is an alkaline compound with a pKa of 8.0 [28]. Because the analytes of 5-FU and 5-FDHU are basic, selecting a pH below its pKa leads to the ionized species. When the organic content increases, its high concentration of buffer makes it prone to precipitate out of solution, thus, the concentration of 10 mM KH_2_PO_4_ is optimal. The analytes 5-FU and its metabolite are polar compounds which can be eluted earlier in 5% organic on the reverse-phase C18 column. To optimize the column separation, several types of column have been tried, such as C18, CN, or HILIC. Finally, the reverse-phase Diamonsil C18 column provides high efficiency, suitability for faster method development and superior batch-to-batch reproducibility. The column with a mobile phase of 5% methanol and 95% 10 mM KH_2_PO_4_ (*v*/*v*) (pH 4.7) achieved acceptable separation of 5-FU and 5-FDHU. 5-FU and 5-FDHU detection was optimized to achieve good sensitivity and peak shape, as well as a relatively short run time.

**Figure 2:**
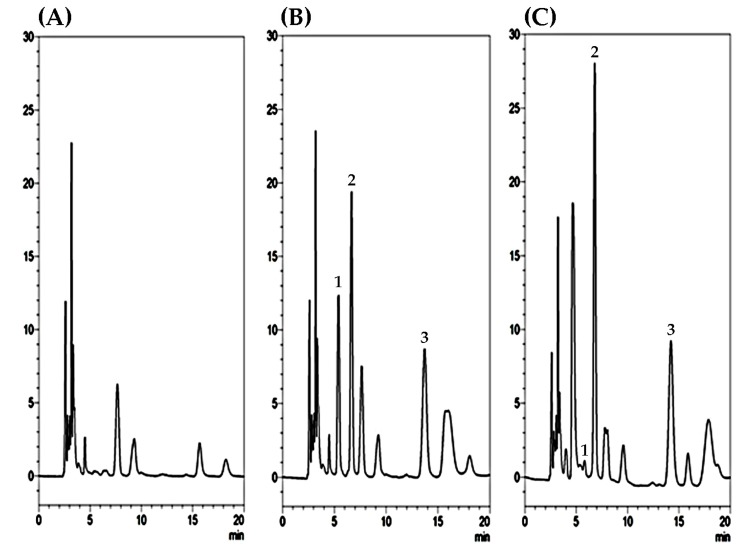
HPLC chromatograms of (**A**) blank plasma samples; (**B**) blank plasma samples spiked with 5-FU (10 µg/mL), 5-FDHU (10 µg/mL), and internal standard (20 µg/mL); and (**C**) blood sample containing 5-FU collected at 30 min after 5-FU (100 mg/kg, iv) administration alone. Peak 1: 5-FDHU with a retention time of 5.8 min. Peak 2: 5-FU with a retention time of 6.8 min. Peak 3: Internal standard amoxicillin with a retention time of 14.2 min.

Regarding the plasma sample preparation, the method of the protein precipitation relied upon the nature of samples and the properties of proteins. According to our experimental experience, methanol precipitation delivered a higher protein recovery and sharp peak compared to acetone precipitation and acetonitrile precipitation. From the practical point of view, the easiest method to perform is methanol precipitation. It usually requires one step, and the supernatant can be obtained after precipitation with methanol. Precipitation with acetone needs larger volumes of organic solvent (at least threefold of sample volume) and it is inconvenient to perform while the volume of original sample is larger than 300 µL. In addition, there was no interference under the present analytical conditions in the range of the retention time of 5-FU and 5-FDHU eluted at 6.8 and 5.8 min, respectively, as shown in Figure 2. Furthermore, selectivity was tested by chromatograms of blank plasma spiked with 5-FU and 5-FDHU standards. The results reveal that good selectivity is achieved under the conditions used in this study. Good linearity was achieved across the range 0.1–50 µg/mL, with all coefficients of correlation greater than 0.995.

### 2.2. Validation of Linearity, Recovery, Precision, Accuracy, and Stability

To validate the analytical system, the limit of detection (LOD) and the lower limit of quantitation (LLOQ) were determined at signal-to-noise ratios (*S*/*N*) of 3 and 10 for the same chromatographic conditions. In this study, the LODs of 5-FU and 5-FDHU were 0.05 µg/mL in plasma. Furthermore, the calibration curves have good linearity (*r*^2^ > 0.999) over a range of 0.1−50 µg/mL. In addition, the mean values of the regression equations for 5-FU and 5-FDHU are *y* = 0.126*x* − 0.015 (*r*^2^ = 1) and *y* = 0.066*x* − 0.001 (*r*^2^ = 1), respectively, in rat plasma. The LLOQ values for this method are 0.1 µg/mL for 5-FU and 0.1 µg/mL for 5-FDHU (Table 1).

The recovery of 5-FU and 5-FDHU from plasma was also estimated at low, medium, and high QC levels (0.5 ng/mL, 5 ng/mL, and 50 ng/mL). After comparing the peak responses of the post-extraction and spiked samples, it was clear that the ratios of the peak responses were within acceptable limits. Absolute 5-FU recovery ranged from 105.1% to 108.4%, 5-FDHU recovery ranged from 99.3% to 105.4% and amoxicillin (internal standard, I.S.) recovery was 100.7% (Table 2). The high reproducibility of the recovery results demonstrated the reliability of the current method for bioanalysis. The extraction recovery of amoxicillin was nearly 100% after sample preparation, which indicates that amoxicillin is suitable as an internal standard for this study.

The determination of the inter-day and intra-day precision and accuracy for this analytical method was conducted by spiking blank plasma with concentrations in the range of the calibration curves. The precision and accuracy for 5-FU and 5-FDHU are summarized in Table 3. As shown in Table 3, six replicates of freshly prepared calibration standard were used to determine the intra-day and inter-day accuracy (% Bias) and precision (% RSD). The intra-day accuracy of 5-FU ranged from −2.92% to 10.6%, with a precision ranging from 0.26% to 6.58%, while the inter-day accuracy ranged from −3.45% to 2.02%, with a precision ranging from 0.05% to 11.5%. The intra-day accuracy of 5-FDHU ranged from −4.68% to 4.41%, with a precision ranging from 0.59% to 8.88%, while the inter-day accuracy ranged from −7.98% to 2.45%, with a precision ranging from 0.09% to 5.70%.

The intra-day and inter-day precision (% RSD) and accuracy (% Bias) values of 5-FU and 5-FDHU in rat plasma were within 15%, except at LLOQ, where the value was within 20%. These validation results show that all replicate measurements were accurate, precise and reproducible for the quantification of 5-FU and 5-FDHU in biological samples (Table 3).

The stability of the analytes was determined through the analysis of low, medium, and high (0.5 µg/mL, 5 µg/mL, 50 µg/mL) concentrations of QC samples, as shown in Table 4. The stability of the three levels of 5-FU and 5-FDHU stock solutions over short-term, autosampler, freeze-thaw and long-term stability ranged from −10.69–4.42% to −12.16–2.68%, respectively, for three replicates. These results indicate that the analytes can be considered stable in rat plasma and post-treatment samples under different storage conditions.

### 2.3. Herbal–Drug Pharmacokinetic Interaction Study

**Figure 3:**
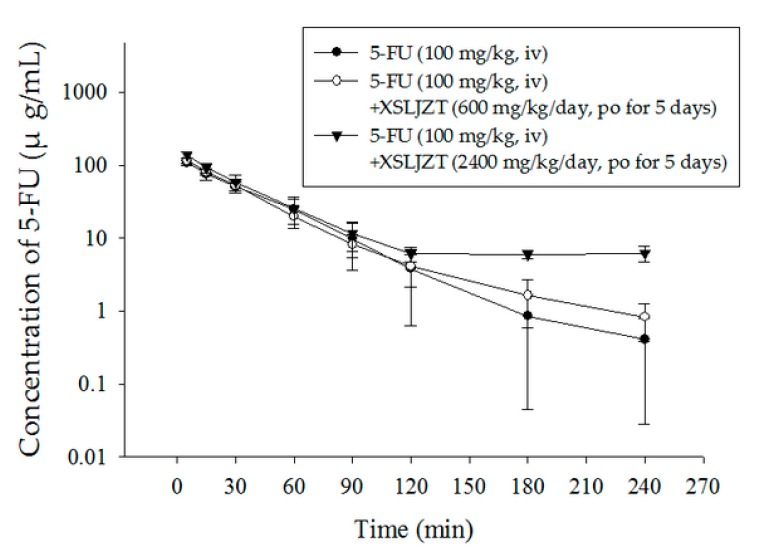
Mean plasma concentration-time curve of 5-FU in rat blood after 5-FU administration (100 mg/kg, iv) alone (●) and 5-FU with a dose of XSLJZT (600 mg/kg/day, po for 5 consecutive days) (○) and XSLJZT (2400 mg/kg/day, po for 5 consecutive days) (▼). po: by mouth; iv: intravenous.

**Figure 4:**
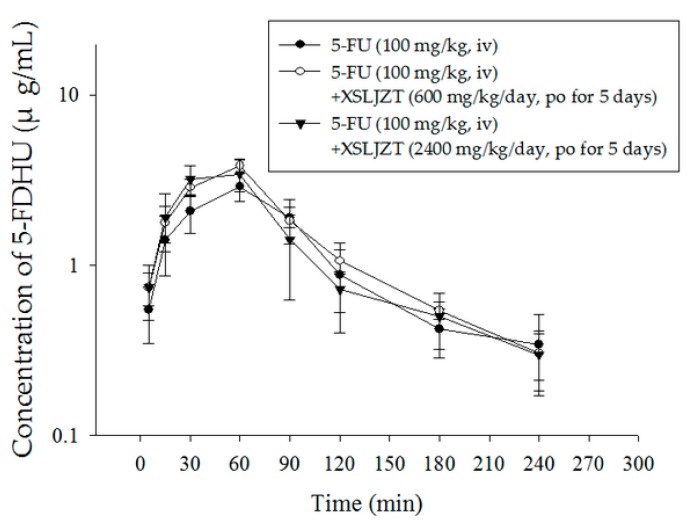
Mean plasma concentration–time curve of 5-FDHU in rat blood after 5-FU administration (100 mg/kg, iv) alone (●), 5-FU with dose of XSLJZT (600 mg/kg/day, po for 5 consecutive days) (○) and XSLJZT (2400 mg/kg/day, po for 5 consecutive days) (▼). po: by mouth; iv: intravenous.

The blood concentration over time of 5-FU and 5-FDHU after two doses of XSLJZT is shown in Figure 3 and Figure 4. The pharmacokinetic parameters of the three groups are summarized in Table 5 and Table 6. The results reveal that the concentration versus time curves (AUC) for 5-FU in plasma are 4527 ± 974 µg/mL, 4640 ± 686 µg/mL, and 6343 ± 1272 µg/mL, following intravenous administration of the three groups, respectively (low dose XSLJZT, high dose XSLJZT, and without XSLJZT). We did not observe extreme variation between the three groups; thus, XSLJZT does not appear to influence the AUC of 5-FU, at both low and high dosages. In contrast, the elimination half-life (*t*_½_) of 5-FU in the 5-FU alone group was 32 ± 12 min, whereas those for rats treated with XSLJZT were 50 ± 15 min, and 50 ± 2 min, respectively. This reveals that concurrent use of 5-FU and XSLJZT prolongs the half-life of 5-FU. The reducing clearance value (CL) and the increasing volume of distribution at steady state (Vss) support this hypothesis, particularly for the high-dose group.

Understanding the mechanism of 5-FU and 5-FDHU in the 5-FU-associated toxicity is crucial for evaluating the herb–drug pharmacokinetics of 5-FU. The empirically determined pharmacokinetic parameters of 5-FDHU are listed in Table 6. The AUC of 5-FDHU in plasma ranged from 321.8 µg/mL to 384.0 µg/mL, and the Cmax and Tmax values of 5-FDHU in the three groups ranged from 2.90 to 3.85 µg/mL and 45 to 60 min, respectively. Furthermore, for assessing elimination, we determined that the mean residence time (MRT) values ranged from 102 to 110 min, the oral CL values from 261 to 320 mL/h/kg, and the half-lives from 68 to 83 min (see Table 6). In summary, there is no significant difference in the pharmacokinetic parameters of 5-FU when used alone, or when used concurrently with XSLJZT.

Our findings are supported by our previous study of 5-FU, in which we found no significant differences resulting from concurrent use with the traditional medicines XSLJZT and JWXYS [10]. Although the results for 5-FU and 5-FDHU did not significantly differ between the 5-FU alone cohort and the group pretreated with a daily dose of XSLJZT (600 mg/kg/day) for five consecutive days, the time-concentration profile indicates that pretreatment with a high-dose of XSLJZT (2400 mg/kg/day) can extend the residence time of 5-FU in blood (that of 5-FDHU is rarely prolonged). Extension of the half-life of 5-FU may be the result of its metabolism, distribution, and elimination. The cytochrome P450 (CYP) superfamily of monooxygenases is primarily associated with drug metabolism in the liver, and CYP1A is involved in the metabolism of 5-FU in rats [29]. A study by Hsueh et al. indicated that 5-FU clearance is markedly increased in CYP1A2-overexpressing rats [30]. Some studies have found that multiple components of the herbal extract can inhibit CYP1A2 activity [31,32]. The ginsenosides Rb1, Rb2, Rc, and Rd, found in Panax ginseng, are known to inhibit the CYP proteins to prolong the half-life of 5-FU following pretreatment with Panax ginseng [33]. In contrast to another study by He et al. 2015, our study found no significant difference in the pharmacokinetic parameters between the group receiving a daily dose of XSLJZT (600 mg/kg/day) with 5-FU, and the 5-FU alone group [34]. However, in the group pretreated with a high dose of XSLJZT (2400 mg/kg/day), 5-FU was maintained at a low concentration for a longer period of time, which might result in an effective concentration of 5-FU, as reported previously [33,35].

In addition to the prolonged duration of 5-FU circulation, another issue is that DPD is the initial rate-limiting enzyme in pyrimidine catabolism, which could induce the degradation of 5-FU into its major catabolite 5-FDHU [36]. Moreover, the relationship between the blood concentration of 5-FU and 5-FDHU are correlated with the toxicity of 5-FU injection in cancer patients [10,37]. Heggie’s study describing the characteristics of 5-FU in intravenously treated patients reported that the half-life and residence time of 5-FU are much shorter than that of its metabolite, 5-FDHU [38]. No significant correlations in 5-FDHU pharmacokinetic parameters were found between the three dosages levels of XSLJZT used in this study, in agreement with Chiang’s studies [10]. In conclusion, the present study provides not only a detailed comparison of the herbal–drug pharmacokinetic interaction of 5-FU and its main metabolite 5-FDHU with XSLJZT in rat blood, but warns that 5-FU-associated toxicity must be considered when coupled with high doses of XSLJZT.

## 3. Materials and Methods

### 3.1. Reagents and Materials

5-FU, 5-FDHU, amoxicillin (internal standard), and urethane were provided by Sigma-Aldrich Chemicals (St. Louis, MO, USA). Potassium dihydrogen phosphate (KH_2_PO_4_), potassium hydroxide (KOH), phosphoric acid (H_3_PO_4_), and methanol of HPLC grade were purchased from E. Merck (Darmstadt, Germany). For all aqueous solutions used in these experiments, deionized water from Millipore (Milford, MA, USA) was used. The pharmaceutical herbal product XSLJZT was manufactured in accordance with Good Manufacturing Practice (GMP) for Chinese Crude Drugs, and was obtained from pharmaceutical companies in Taiwan; this compound has been approved for medicinal use in patients. The pharmaceutical herbal product XSLJZT was purchased from Sun-Ten Pharmaceutical Co., Ltd. (Taipei, Taiwan).

5-FU and 5-FDHU were dissolved in methanol to produce a standard solution (1 mg/mL) and then diluted in Eppendorf vials to make a stock solution (10 µg/mL). The working solution was prepared by diluting the stock solution in 50% (*v*/*v*) methanol to obtain the following concentrations: 0.1, 0.5, 1, 5, 10 and 50 µg/mL. All stock solutions were stored in the dark at −20 °C.

### 3.2. Instrumentation and HPLC–UV Conditions

The HPLC system consisted of chromatographic pumps (LC-20AT; Shimadzu Co., Kyoto, Japan), an autosampler (SIL-20AC; Shimadzu Co.), and a photodiode array detector (SPDM20A; Shimadzu Co.). All analytical samples were separated using a reverse-phase Diamonsil C18 column (250 mm × 4.6 mm internal diameter; particle size 5 µm; Dikma, Lake Forest, CA, USA). The mobile phase for HPLC analysis consisted of two solvent compositions: 10 mM potassium dihydrogen phosphate (KH_2_PO_4_) and methanol (95:5, *v*/*v*). The 10 mM KH_2_PO_4_ solution was adjusted to pH 4.7 using phosphoric acid or potassium hydroxide. The flow rate for the mobile phase was set at 1 mL/min. The temperature in the autosampler was set at 4 °C, the analytical volume was 10 µL of each sample, the UV–vis detector scanned from 200 to 500 nm, and the chromatographic profiles were monitored at 215 nm for 5-FU and 5-FDHU.

### 3.3. Preparation of 5-FU and 5-FDHU Plasma Extraction

Samples were prepared as follows. First, 50 µL of rat plasma was mixed with 10 µL of internal standard (amoxicillin) solution and 140 µL methanol for protein precipitation. The samples were vortex-mixed for 5 min and centrifuged at 13,000× *g* at 4 °C for 10 min. The supernatants were purified through a 0.22 µm filter prior to HPLC–UV analysis.

### 3.4. Method Validation

#### 3.4.1. Calibration Curves

Calibration curves were generated by spiking blank rat plasma with different concentrations of the working solutions. The calibration curves ranged from 0.1 to 50 µg/mL for the blood. The linearity of the assay was assessed using the coefficient of determination (*r*^2^) for the calibration curve, which should be greater than 0.995. The limit of detection (LOD) was determined at the concentration that generated a signal-to-noise ratio of 3, and the lower limit of quantification (LLOQ) was defined as the lowest concentration of the linear regression that yielded a signal-to-noise ratio of 10.

#### 3.4.2. Extraction Recovery

The extraction recovery was calculated using two sets of samples. 5-FU and 5-FDHU were diluted to 0.5, 5 and 50 µg/mL in the mobile phase. The two sets were as follows:

Set 1 The stock solutions of 5-FU and 5-FDHU were mixed with 10 µL of amoxicillin (I.S.) solution and diluted to 0.5, 5 and 50 µg/mL in the mobile phase.
Set 2 A total of 10 µL of standard solution was added to 50 µL of blank plasma, 10 µL of amoxicillin (I.S.) solution and 130 µL of methanol and prepared as described in the sample preparation section. Pre-extraction samples of 5-FU and 5-FDHU were prepared and used for HPLC–UV analysis. The recovery was calculated as the peak area of Set 2 divided by the peak area of Set 1.

#### 3.4.3. Evaluation of Accuracy and Precision

The accuracy and precision evaluation was based on the Food and Drug Administration (FDA) Guidelines [39]. The accuracy was estimated as bias (%) = (observed concentration − nominal concentration) × 100/nominal concentration. The precision was calculated by relative standard deviation, RSD % = (SD) × 100/observed concentration. The calibration of six replications on the same day (intra-day) and six successive days (inter-day) was carried out to verify the accuracy and precision of the method. Samples of 5-FU and 5-FDHU were prepared at concentrations of 0.1, 0.5, 1, 5, 10 and 50 µg/mL. The calibration curve was characterized using the peak area ratio of 5-FU and 5-FDHU hydrochloride versus the concentration.

### 3.5. Stability Evaluation

The stability of 5-FU and 5-FDHU were evaluated using the following methods, in accordance with Food and Drug Administration guidelines [39]:

(1) Short-term: The samples were stored at room temperature (25 ± 3 °C) for 4 h before analysis.
(2) Post-preparative: The samples were kept at 8 °C for 8 h in an autosampler before analysis.
(3) Freeze and thaw: The samples were stored at −20 °C for 24 h and then thawed at room temperature. The freeze and thaw cycle was repeated three times.
(4) Long-term: The samples were kept at −20 °C for 30 days in darkness before analysis.

Concentrations of 0.5, 5 and 50 µg/mL of 5-FU and 5-FDHU were selected to measure stability. The relative error between freshly prepared samples and stored samples was calculated to determine stability. The limitation of sample stability was defined as within ±15%, and LLOQ values were less than ±20%.

### 3.6. Experimental Animals

The protocol was reviewed and approved by the Institutional Animal Experimentation Committee of National Yang-Ming University, Taipei, Taiwan and by the Institutional Animal Care and Use Committee (IACUC; Approval number 106DN22; Approval date 01/08/2017). Male Sprague Dawley rats (250–280 g) were provided by the Laboratory Animal Center at National Yang-Ming University (Taipei, Taiwan). The animals had free access to water and food (laboratory rodent diet 5P14, PMI Feeds, Richmond, IN, USA) ad libitum and were housed in a pathogen-free environment with a 12 h light/dark cycle. All animal experiments followed the guidelines and procedures for the care of laboratory animals in National Yang-Ming University. The study was carried out over a 5 day period, and the rats were randomly separated into 3 treatment groups: Group (1), 5-FU (100 mg/kg, iv) administered alone with deionized water for 5 consecutive days; group (2), a pretreated normal dose of XSLJZT (600 mg/kg/day, po) for 5 consecutive days and on the 5th day + 5-FU (100 mg/kg, iv); and group (3), a pretreated high dose of XSLJZT (2400 mg/kg/day, po) for 5 consecutive days and on the 5th day + 5-FU (100 mg/kg, iv).

### 3.7. Drug Administration

In this study, the dose used for rats was calculated from the human dosage according to the body surface area normalization method. For humans, the initial treatment and maintenance therapy in a clinical setting requires a 5-FU dose of 600 mg/m^2^; by using the formula, the equivalent dosage of 5-FU in rats is 100 mg/kg [40,41]. A previous study compared the dose-normalized area under the curve (AUC) at different intravenous doses of 5-FU in rats and found that a dose of 100 mg/kg was suitable for the determination of 5-FU pharmacokinetic parameters [42]. Pharmaceutical grade herbal XSLJZT powder was dissolved in deionized water at a concentration of 0.25 g/mL for oral administration to the rats via gavage. A daily dose of XSLJZT herbal powder for adults is 6 g taken once a day, which is equivalent to 600 mg/kg/day for rats. This dosage is referred to as the normal dose in this study. However, the dose of XSLJZT taken is dependent on symptoms, so a high dose (2400 mg/kg/day) of XSLJZT was also applied to determine the herb–drug interaction in detail. Rats in group 1 were initially anesthetized through intraperitoneal injection with an anesthetic mixture (10 mL/kg, ip—intraperitoneal) of urethane (1 g/kg) and distilled water, and then given 5-FU (100 mg/kg, iv) alone through the femoral vein. Rats in groups 2 and 3 were pretreated with different oral doses of XSLJZT for 5 consecutive days, and on the 5th day after pretreatment, were given XSLJZT for 1 h; the rats were anesthetized by intraperitoneal injection of the anesthetic mixture (10 mL/kg, ip) prior to surgery. Finally, 5-FU (100 mg/kg, iv) was injected into the femoral vein, and the rats were maintained under anesthesia for the duration of the experiment. Blood samples were collected at 5, 15, 30, 60, 90, 120, 180 and 240 min. At the end of the experiment, the rats were euthanized through carbon dioxide overdose while under anesthesia.

### 3.8. Data Analysis

The pharmacokinetic parameters were determined by analyzing each individual set of data with a non-compartmental model using the software WinNonlin Standard Edition Version 1.1 (Scientific Consulting Inc., Apex, NC, USA). The pharmacokinetic parameters include the initial drug concentration of 5-FU (*C*_0_), the maximum concentration and time of 5-FDHU (*C*_max_, *t*_max_), the area under the concentration versus time curve (AUC), the clearance (CL), the elimination half-life (*t*_½_), the volume of distribution at steady state (Vss), and the mean residence time (MRT). Statistical analyses were performed using analysis of variance function in SPSS 18.0 (SPSS Inc., Chicago, IL, USA) and SigmaPlot 10.0 software. All data are expressed as the mean ± standard deviation (SD). One-way ANOVA was used for the comparison between groups, and statistically significant differences were defined as * *p* < 0.05 or ** p < 0.01.

## 4. Conclusions

In recent years, the concomitant use of Oriental and Western medicine for the treatment of cancer patients has increased. Many studies have confirmed that alternative treatments, such as traditional Chinese medicine (TCM), can reduce the adverse effects of cancer chemotherapy and improve the quality of life of the patient. Therefore, understanding the interactions between herbal medicines and modern drugs is extremely important. The validation analytical method of 5-FU and 5-FDHU is one of the highlights of our study, and we have also applied the developed analytical methods to herb–drug pharmacokinetic interaction with herbal medicine in rat. This study demonstrates that the herbal formulation of XSLJZT has no significant herb–drug interaction with 5-FU and its major metabolite 5-FDHU in rat blood under a daily dosage regimen. From the pharmacokinetics viewpoint, the coadministration of 5-FU and XSLJZT is likely to be safe.

## Figures and Tables

**Table 1:** Linear ranges, calibration curves, correlation coefficients (*r*^2^), and detection limits of 5-FU and 5-FDHU.

Compounds	Linear Ranges (µg/mL)	Calibration Curves	*r*^2^	LLOQ (µg/mL)	LOD (µg/mL)
5-FU	0.1–50	*y* = 0.126*x* − 0.015	1.0000	0.1	0.05
5-FDHU	0.1–50	*y* = 0.066*x* − 0.001	1.0000	0.1	0.05

LLOQ: Lower limit of quantification at a signal-to-noise ratio (*S*/*N*) of 10; LOD: Limit of detection was determined at a signal-to-noise ratio (*S*/*N*) of 3.

**Table 2:** Extraction recoveries of 5-FU, 5-FDHU, and amoxicillin (I.S.) in rat plasma.

Con. (µg/mL)	Spiked in the Mobile Phase (Set 1)	Spiked Before Extraction (Set 2)	Recovery (%)
5-FU			
0.5	12,884 ± 492	13,876 ± 897	107.8 ± 0.09
5	149,388 ± 5857	161,755 ± 3696	108.4 ± 0.07
50	1,487,375 ± 31,269	1,562,657 ± 42,890	105.1 ± 0.05
5-FDHU			
0.5	8281 ± 520	8699 ± 141	105.4 ± 0.09
5	85,864 ± 2239	85,206 ± 4730	99.29 ± 0.06
50	871,855 ± 13,873	887,507 ± 50,640	101.7 ± 0.04
Amoxicillin (I.S.)			
20	265,553 ± 4096	267,379 ± 10,503	100.7 ± 0.05

Data are expressed as the means ± S.D. The recovery (%) = (the peak area of Set 2/the peak area of Set 1) × 100. I.S.: internal standard.

**Table 3:** Inter-day and intra-day assay precision (% RSD) and accuracy (% Bias) values for the HPLC–UV method for the quantification of 5-FU and 5-FDHU in rat plasma.

Nominal Con. (µg/mL)	Intra-Day (*n* = 6)			Inter-Day (*n* = 6)		
	Observed Con. (µg/mL)	Accuracy Bias (%)	Precision RSD (%)	Observed Con. (µg/mL)	Accuracy Bias (%)	Precision RSD (%)
5-FU						
0.1	0.111 ± 0.01	10.6	6.58	0.102 ± 0.01	2.02	11.5
0.5	0.528 ± 0.04	5.69	1.03	0.487 ± 0.04	−2.69	7.35
1	0.971 ± 0.03	−2.92	2.70	0.966 ± 0.03	−3.45	3.32
5	5.018 ± 0.05	0.37	1.08	4.965 ± 0.12	−0.70	2.43
10	10.02 ± 0.12	0.21	1.16	10.04 ± 0.12	0.40	1.17
50	50.15 ± 0.13	0.30	0.26	49.98 ± 0.02	−0.04	0.05
5-FDHU						
0.1	0.101 ± 0.01	0.60	8.88	0.092 ± 0.01	−7.98	5.70
0.5	0.489 ± 0.04	−2.23	7.43	0.503 ± 0.01	0.51	1.74
1	1.044 ± 0.04	4.41	3.44	1.025 ± 0.04	2.45	3.61
5	4.766 ± 0.25	−4.68	5.20	4.867 ± 0.17	−2.65	3.50
10	10.26 ± 0.31	2.61	3.03	10.11 ± 0.19	1.05	1.84
50	50.16 ± 0.30	0.31	0.59	49.99 ± 0.04	−0.02	0.09

Data are expressed as the means ± SD. Precision (%RSD) = SD/C_obs_ × 100. Accuracy (%Bias) = (C_observation_ − C_nominal_)/C_nominal_ × 100.

**Table 4:** Stability of 5-FU and 5-FDHU in rat plasma QC samples.

Analytes/Spiked Concentration (µg/mL)	Short-Term Stability	Autosampler Stability	Freeze-Thaw Stability	Long-Term Stability
5-FU				
0.5	2.95 ± 0.02	4.42 ± 0.04	−6.10 ± 0.06	−10.69 ± 0.01
5	1.17± 0.04	2.93 ± 0.07	−2.64 ± 0.07	−6.74 ± 0.06
50	0.06 ± 0.03	−0.34 ± 0.05	−1.41± 0.03	−2.53 ± 0.08
5-FDHU				
0.5	2.53 ± 0.05	2.68 ± 0.05	−5.24 ± 0.05	−12.16 ± 0.05
5	1.78 ± 0.05	−2.29 ± 0.02	−3.85 ± 0.02	−4.53 ± 0.05
50	0.24 ± 0.11	−0.23 ± 0.12	−1.68 ± 0.14	−1.96 ± 0.03

Data are expressed as the mean ± SD (*n* = 3). The stability (%) was calculated as follows: Stability (%) = (C_post_ QC − C_pre_ QC) × 100/C_pre_ QC. Short-term stability: Room temperature for 4 h; autosampler stability: 8 °C for 8 h at the autosampler; freeze-thaw stability: Three freeze-thaw cycles; long-term stability: Storage at −20 °C for 30 days. C_post_ QC: the post-concentration of quality control. C_pre_ QC: the pre-concentration of quality control.

**Table 5:** Pharmacokinetic parameters of 5-FU (100 mg/kg, iv) in rat.

Parameter	Unit	5-FU (100 mg/kg, iv)	5-FU + XSLJZT (600 mg/kg/day, po)	5-FU + XSLJZT (2400 mg/kg/day, po)
AUC	min μg/mL	4527 ± 974	4640 ± 686	6343 ± 1272 *
*C*_0_	μg/mL	129.4 ± 14.4	140.9 ± 13.6	150.9 ± 11.5
*t*_½_	min	32 ± 12.0	50 ± 15.1 *	50 ± 1.51 *
Cl	mL/min/kg	22.87 ± 4.39	21.97 ± 3.43	16.32 ± 3.39 *
Vss	mL/kg	770.2 ± 52.1	815.4 ± 115	1084 ± 188 **
MRT	min	35 ± 7.06	38 ± 6.91	67 ± 4.76 **

AUC, area under the concentration versus time curve; *C*_0_, initial drug concentration; Cl clearance; *t*_½_, elimination half-life; Vss, volume of distribution at steady state; MRT, mean residence time. Cl = Dose/AUC; Vss = Dose/C_0_; MRT = AUMC/AUC. Data are expressed as the means ± SD (*n* = 6). * *p* < 0.05 and ** *p* < 0.01 compared with that of the 5-FU alone group. po: by mouth; iv: intravenous.

**Table 6:** Pharmacokinetic parameters of 5-FDHU is a metabolite of 5-FU.

Parameter	Unit	5-FU (100 mg/kg, iv)	5-FU + XSLJZT (600 mg/kg/day, po)	5-FU + XSLJZT (2400 mg/kg/day, po)
AUC	min μg/mL	321.8 ± 59.7	384.0 ± 19.9	354.2 ± 45.4
*C*_max_	μg/mL	2.904 ± 0.54	3.849 ± 0.36	3.688 ± 0.34
*T*_max_	min	60	60	45 ± 16.4
*t*_½_	min	71 ± 9.62	68.00 ± 19.9	83 ± 32.4
Cl	mL/min/kg	320.0 ± 61.2	261.0 ± 13.4	286.8 ± 42.8
MRT	min	110 ± 18.5	102 ± 15.0	105 ± 18.7

AUC, the area under the concentration versus time curve; *C*_max_, the maximum concentration; *T*_max_, the time taken to reach the maximum concentration; Cl, the clearance; *t*_½_, the elimination half-life; MRT, mean residence time. Cl = Dose/AUC; MRT = AUMC/AUC. Data are expressed as the means ± SD (*n* = 6). * *p* < 0.05 and ** *p* < 0.01 compared with that of the 5-FU alone group. po: by mouth; iv: intravenous.
